# Microglial depletion does not impact alpha-synuclein aggregation or nigrostriatal degeneration in the rat preformed fibril model

**DOI:** 10.21203/rs.3.rs-2890683/v1

**Published:** 2023-05-04

**Authors:** Anna C. Stoll, Christopher J. Kemp, Joseph R. Patterson, Michael Kubik, Nathan Kuhn, Matthew Benskey, Megan F. Duffy, Kelvin Luk, Caryl E. Sortwell

**Affiliations:** Michigan State University; Michigan State University; Michigan State University; Michigan State University; Michigan State University; Michigan State University; Michigan State University; University of Pennsylvania Perelman School of Medicine; Michigan State University

**Keywords:** PLX3397, Neuroinflammation, Parkinson’s disease, Synucleinopathy, Major-histocompatibility complex-II, Neurodegeneration, Colony stimulating factor-1 receptor inhibition, Substantia nigra

## Abstract

**Background:**

Parkinson’s disease (PD) is a neurodegenerative disorder that is characterized by the presence of proteinaceous alpha-synuclein (α-syn) inclusions (Lewy bodies), markers of neuroinflammation and the progressive loss of nigrostriatal dopamine (DA) neurons. These pathological features can be recapitulated in vivo using the α-syn preformed fibril (PFF) model of synucleinopathy. We have previously described the time course of microglial major-histocompatibility complex-II (MHC-II) expression and alterations in microglia morphology in the PFF model in rats. Specifically, the peaks of α-syn inclusion formation, MHC-II expression, and reactive morphology in the substantia nigra pars compacta (SNpc) all occur two months post PFF injection, months before neurodegeneration occurs. These results suggest that activated microglia may contribute to neurodegeneration and could represent a potential target for novel therapeutics. The goal of this study was to determine whether microglial depletion could impact the magnitude of α-syn aggregation, nigrostriatal degeneration, or related microglial activation during the α-syn PFF model.

**Methods:**

Male Fischer 344 rats were injected intrastriatally with either α-syn PFFs or saline. Rats were continuously administered Pexidartinib (PLX3397B, 600mg/kg), a colony stimulating factor-1 receptor (CSF1R) inhibitor, to deplete microglia for a period of either two or six months.

**Results:**

PLX3397B administration resulted in significant depletion (45–53%) of ionized calcium-binding adapter molecule 1 immunoreactive (Iba-1ir) microglia within the SNpc. Microglial depletion did not impact accumulation of phosphorylated α-syn (pSyn) within SNpc neurons and did not alter pSyn associated microglial reactivity or expression of MHC-II. Further, microglial depletion did not impact SNpc neuron degeneration. Paradoxically, long term microglial depletion resulted in increased soma size of remaining microglia in both control and PFF rats, as well as expression of MHC-II in extranigral regions.

**Conclusions:**

Collectively, our results suggest that microglial depletion is not a viable disease-modifying strategy for PD and that partial microglial depletion can induce a heightened proinflammatory state in remaining microglia.

## Background

Parkinson’s Disease (PD), the second most common neurodegenerative disease, affects around 1 million people in the USA with 60,000 newly diagnosed people each year (1). Pathologically, PD is characterized by the presence of proteinaceous alpha-synuclein (α-syn) inclusions (Lewy bodies) and the progressive loss of the nigrostriatal dopamine (DA) neurons (2). While the exact cause of PD is still unknown, mounting evidence has suggested that neuroinflammation, mediated by microglia, may play a significant role in PD progression and neuropathology. Microglia, have many roles in helping maintain healthy homeostasis in the brain, including synaptic pruning, neurogenesis, and neuronal surveillance (3–5). However, microglia are main players in the immune response to an insult and allow for the bridging of the innate and adaptive immune system (6,7). Analysis of postmortem PD brain show increased inflammatory markers, including increases in cells immunoreactive for ionized calcium binding adaptor molecule 1 (Iba1), human leukocyte antigen (HLA-DR), and phagocytic marker CD68 in the vicinity of Lewy pathology, specifically the substantia nigra (SN) (8–12). Patients with PD have elevated proinflammatory cytokines (i.e., interleukin 1-beta, interleukin-6, interferon gamma, and tumor necrosis factor-alpha) in their cerebral spinal fluid (CSF) and plasma, all produced by microglia and immune cells (13–16).

These pathological hallmarks of PD; α-syn inclusions, loss of dopaminergic neurons and neuroinflammation, can be recapitulated *in vivo* using the α-syn preformed fibril (PFF) model of synucleinopathy (17–20). We have previously described the time course of the accumulation of phosphorylated α-syn (pSyn) inclusions, nigrostriatal degeneration, and microglial reactivity in the rat PFF model (17,21). Specifically, the peak of pSyn inclusion formation, number of major-histocompatibility complex-II immunoreactive (MHC-IIir) microglia and microglial soma size in the substantia nigra pars compacta (SNpc) occurs two months post intrastriatal PFF injection, months before the neurodegeneration phase occurring at 5–6 months (21,22). Of importance, a localized subpopulation of MHC-IIir microglia is observed immediately adjacent to nigral pSyn inclusions, with the number of reactive microglia dependent on nigral inclusion load (21). These results, along with results from other laboratories (23–26) suggest that pSyn inclusions are immunogenic, provoking a microglial proinflammatory response that has the potential to contribute to ultimate nigrostriatal neurodegeneration. Thus, therapeutic strategies that target and attenuate this microglial response to pathological α-syn may have potential to slow disease progression.

Pexidartinib (PLX3397B; Plexxikon inc.), a selective tyrosine kinase inhibitor, targets the macrophage (i.e. microglia) colony stimulating factor 1 receptor (CSF1R). The CSF1R is required for the activation, proliferation, and survival of microglia and, when inhibited, leads to microglial death resulting in microglial depletion within the brain parenchyma (27). Microglial depletion has previously been analyzed in mouse models of disease to understand the role microglia may play in disease progression (28–30). However, microglia are required to maintain healthy brain homeostasis and as such, complete microglia depletion may not be a viable therapeutic strategy. Therefore, in the present study we examined the effect of partial microglia depletion on α-syn aggregation and neurodegeneration within the rat PFF model. We demonstrate that significant, partial microglia depletion (36–46%) does not affect pSyn inclusion accumulation in the SNpc 2-months following α-syn PFF injection or the inclusion-associated microglial response of increased microglial soma size and expression of MHC-II. Further, microglial depletion did not impact PFF induced degeneration of tyrosine hydroxylase immunoreactive (THir) neurons in the SNpc. Surprisingly, long term microglial depletion was associated with increased microglial soma size in remaining microglia as well as expression of MHC-II in extranigral regions. Our results do not support microglial depletion as a disease modifying strategy for PD and instead suggest that long term microglial depletion may be detrimental through induction of a proinflammatory phenotype in remaining microglia.

## Methods

### Experimental Overview

**Figure 1:**
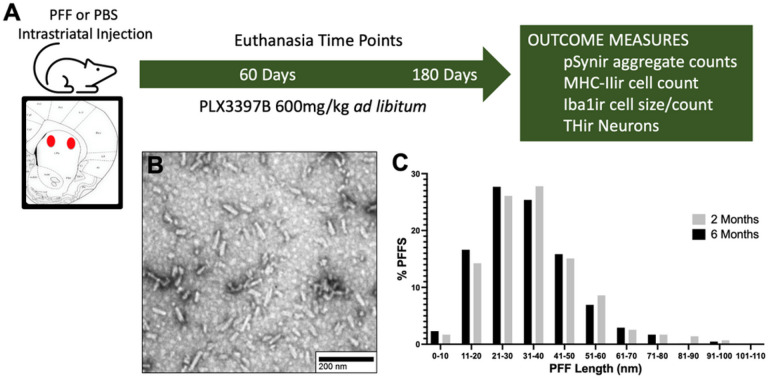
Experimental Design and PFF Size Distribution **A**: Male Fischer 344 rats (3-months of age) received two intrastriatal injections of sonicated mouse alpha-synuclein preformed fibrils (α-syn PFFs) or phosphate buffered saline (PBS). Rats were fed Pexidartinib (PLX3397B) or control chow ad libitum starting on the day of surgery until euthanasia at two or six months post-surgery. Brains were collected for postmortem endpoints including quantification of a-syn phosphorylated at serine 129 immunoreactive (pSynir) neurons, major histocompatibility complex II immunoreactive (MHC-IIir) cells, tyrosine hydroxylase immunoreactive (THir) neurons, and ionized calcium-binding adaptor molecule 1 immunoreactive (Iba1ir) microglia count and size, in the substantia nigra pars compacts (SNpc). **B**: Representative electron micrograph of sonicated α-syn fibrils. **C**: Size distribution of ~650 sonicated fibrils prior to injection (mean fibril size- 2 months: 35.9 ± 0.06 nm, 6-months: 34 ± 0.57 nm).

Rats received unilateral intrastriatal injections of either mouse α-syn PFFs or an equal volume of phosphate buffered saline (PBS) and were fed the CSF1R inhibitor PLX3397B or control chow for a period of either 60 or 180 days. At the conclusion of the experiment rats were euthanized and brain tissue analyzed. Figure 1Aillustrates the experimental design.

### Rats

Three-month old, male Fischer 344 rats (Charles River) were housed, 2–3 per cage, at the Grand Rapids Research Center vivarium which is fully approved through the Association for Assessment and Accreditation of Laboratory Animal Care (AAALAC). Rats were housed in a room with a 12-hour light/dark cycle and provided food and water *ad libitum*. All procedures were done in accordance with the guidelines set by the Institutional Animal Care and Use Committee (IACUC) of Michigan State University.

### α-syn PFF Preparation and Fibril Measurements

α-syn PFFs were generated from wild-type-full length, recombinant mouse α-syn monomers as previously described (18,31–33). Quality control was completed on full length fibrils to ensure fibril formation (transmission electron microscopy), amyloid structure (thioflavin T assay), pelletability as compared to monomers (sedimentation assay), and low endotoxin contamination (*Limulus* amebocyte lysate assay; <0.5 endotoxin units/mgof total protein). On surgery day, PFFs were thawed to room temperature and diluted to 4 μg/μl in sterile Dulbecco’s PBS and sonicated with an ultrasonic homogenizer (300 VT; Biologics, Inc.) for 60– 1s pulses, pulser set at 20% and power output at 30%. A sample of sonicated PFFs were prepared on Formvar/carbon-coated copper grids (EMSDIASUM, FCF300-Cu). Fibrils were then imaged with a JEOL JEM-1400+ transmission electron microscope (34). The length of ~650 fibrils was determined using ImageJ 1.53K (Wayne Rasband and contributors, National Institutes of Health, USA) (Figure 1B,C). The mean length for the 2-month surgical cohort was 35.9 ± 0.06 nm and for the 6-month surgical cohort was 34 ± 0.57 nm. Fibril length < 50 nm is required for efficient seeding of endogenous α-syn inclusions (35).

### Stereotaxic Injections

Unilateral intrastriatal α-syn PFF injections were conducted as previously described (17). Rats were anesthetized with isoflurane (5% induction and 1.5% maintenance) and received unilateral intrastriatal injections to the left hemisphere (2 × 2 μl, AP +1.6, ML +2.0, DV −4.0; AP +0.1, ML +4.2, DV −5.0, AP and ML coordinates relative to Bregma, DV coordinates relative to dura). PFFs (4 μg/μl; 16 μg total) or an equal volume of PBS were injected at a rate of 0.5 μl/min with a pulled glass capillary tube attached to a 10 μl Hamilton syringe (34). To avoid PFF displacement, the needle was left in place for 1 minute following injection, retracted 0.5 mm and left for 2 minutes before fully retracted. All animals received analgesic (1.2 mg/kg of sustained release buprenorphine) after surgery and were monitored until euthanasia.

### Pexidartinib Dosing

Pexidartinib chow was generously provided by Plexxikon, Inc. Rats were fed Pexidartinib chow (PLX3397B, 600mg/kg; Plexxikon Inc.; Research Diets Inc.) or control chow *ad libitum* for either 60 or 180 days starting on the day of PFF injections. Rat weights and collective cage food intake was tracked weekly (Supplemental Figures 1A-B, 2A-B).

### Euthanasia

Rats were euthanized at 60 days (peak pSyn accumulation in the SNpc) or 180 days (peak nigral degeneration) post-surgery, pathological intervals that have been previously identified in this model (17,21,36). Rats were given a 30 mg/kg pentobarbital injection (i.p.) (Euthanasia-III Solution, MED-PHARMEX, Inc.) and perfused intracardially with heparinized 0.9% saline. Livers were removed and weighed (Supplemental Figures 1C, 2C). Brains were removed and post-fixed in 4% paraformaldehyde (PFA) for one week and then transferred to 30% sucrose in 0.1M phosphate buffer until sunk. Brains were frozen on dry ice and cut at 40 μm on a sliding microtome, sections were stored in cryoprotectant (30% sucrose, 30% ethylene glycol, in 0.1M Phosphate Buffer (PB), pH 7.3) at −20°C.

### Immunohistochemistry

Free floating sections were washed 4 × 5minutes in 0.1M tris buffered saline (TBS) containing 0.5% Triton-X100 (TBS-Tx), quenched in 3% H_2_O_2_ for 1 hour, blocked in 10% normal goat serum (NGS) in TBX-Tx, and incubated overnight in primary antibody in 1% NGS/TBS-Tx at 4°C on a shaker. Primary antibodies used included: mouse anti-α-syn phosphorylated at serine 129 (pSyn) (1:10,000; Abcam, AB184674), mouse anti-tyrosine hydroxylase (TH) (1:4000; Millipore, MAB318), rabbit anti-ionized calcium binding adaptor molecule 1 (Iba1) (1:1,000; Wako, 019–09741), mouse anti-major histocompatibility complex-II (MHC Class II RT1B clone OX-6) (1:2,000; BioRad, MCA46G). Sections were washed in TBS-Tx and then incubated for 2-hours at room temperature with biotinylated secondary antibodies in 1% NGS/TBS-Tx. Secondary antibodies used included: goat anti-mouse IgG (1:500; Millipore, AP124B), goat anti-rabbit IgG (1:500, Millipore, AP132B), and horse anti-mouse IgG rat preabsorbed (for TH; 1:500; Vector Laboratories, BA-2001). Sections were washed 4 × 5 minutes in TBS-Tx and incubated in standard avidin-biotin complex detection kit (ABC, Vector Laboratories, PK-6100). Visualization for pSyn was done using 2.5 mg/ml nickel ammonium sulfate hexahydrate (Fisher, N48–500), 0.5 mg/ml diaminobenzidine (Sigma-Aldrich, D5637), and 0.03% H_2_O_2_ in TBS-Tx. TH was visualized with 0.5 mg/ml diaminobenzidine (Sigma-Aldrich, D5637), and 0.03% H_2_O_2_ in TBS-Tx. MHC-II was visualized using Vector ImmPACT DAB (brown) Peroxidase kit (Vector Laboratories; SK-4105). Iba1 was visualized with ImmPACT VIP (purple) Peroxidase Kit (Vector Laboratories; SK-4605). Sections were mounted, allowed to dry, rehydrated, then dehydrated in ascending ethanol washes and cleared with xylene before cover slipping using Epredia Cytoseal-60 (Thermo-Fischer, 22–050-262). pSyn sections were counterstained with cresyl violet before dehydration.

### Immunofluorescence

Free floating sections were washed 5 × 5 minutes in TBS-Tx, blocked in 10% NGS in TBX-Tx, then incubated overnight in primary antibodies in 1% NGS/TBS-Tx at 4°C on a shaker. Primary antibodies used included: mouse anti-pSyn (1:10000; Abcam, AB184674) and rabbit anti-Iba1 (1:1,000; Wako, 019–09741). Sections were washed in TBS-Tx and then incubated for 2-hours, in the dark, at room temperature, with fluorescent conjugated secondary antibodies in 1%NGS/TBS-Tx. Secondary antibodies used included: Alexa Fluor 568 goat anti-mouse IgG (1:500, Invitrogen, A-11004), and Alexa Fluor 647 goat anti rabbit IgG (1:500, Invitrogen, A32733). Sections were then rinsed 5 × 5 minutes in TBS-Tx, incubated 1 × 5 minutes in 4’,6-Diamidino-2-Phenylindole, Dihydrochloride (DAPI) made in TBS-Tx (1:10,000, Invitrogen, D1306) and placed back in TBS-Tx for mounting. Sections were mounted and cover-slipped with VECTASHIELD Vibrance antifade mounting medium (Vector Laboratories, H-1700) and kept in the dark until imaging utilizing the Zeiss Axioscan.Z1 scanning microscope.

### Total Enumeration for pSyn and MHC-II

Due to heterogeneity in the distribution of both pSyn and MHC-II immunoreactive profiles within the SN, total enumeration rather than stereological counting frames was used for quantification. The investigator was blinded to treatment groups. Total enumeration of pSyn immunoreactive (pSynir) neurons and MHC-IIir cells was conducted utilizing Microbrightfield Stereoinvestigator (MBF Bioscience). Sections containing the SN pars compacta (SNpc, 1:6 series) were used. Contours were drawn around the SNpc at 4X, a 20x magnification was then used for identification and counting. Counts represent the raw total number multiplied by six. Data are reported as total estimates of pSynir neurons or MHC-IIir cells in each hemisphere.

### Stereological Assessment of Nigral TH Immunoreactive Neurons

The number of THir neurons in the ipsilateral and contralateral SNpc was estimated using unbiased stereology with the optical fractionator principle. The investigator was blinded to treatment groups. Using a Nikon Eclipse 80i microscope, Retiga 4000R camera (QImaging) and Microbrightfield StereoInvestigator software (Microbrightfield Bioscience, Williston, VT), THir neuron quantification was completed by drawing a contour around the SNpc borders using the 4X objective on every sixth and counting neurons according to stereological principles at 60X magnification. Briefly, counting frames (50 μm × 50 μm) were systematically and randomly distributed over a grid (183 μm × 122 μm) overlaid on the SNpc. A coefficient of error < 0.10 was accepted. Data are reported as total estimate of THir neurons in each hemisphere.

### Microglia Soma Size and Number

Nigral sections were fluorescently labeled for pSyn and Iba1. The investigator was blinded to treatment groups. Utilizing the Zeiss Axioscan.Z1 scanning microscope, Z-Stacks images at 20X were obtained and three consecutive nigral sections representing the sections with the highest number of pSynir neurons were analyzed with Nikon Elements AR (Version 4.50.00, Melville, NY). All Iba1ir soma were outlined, excluding processes, and the number of individual microglial objects calculated. Data for soma size is reported as the number of pixels per outlined microglia soma. The HALO^®^ (Indica Labs) image analysis module “Area quantification v1.0 for brightfield” was used to calculate total pSyn signal in the striatum and MHC-II signal in the mesencephalon.

### Statistical Analysis

All statistical tests were completed using GraphPad Prism software (version 9, GraphPad, La Jolla, CA). Outliers were assessed via the absolute deviation from the median method (37) utilizing the very conservative difference of 2.5X median absolute deviation as the exclusion criteria. Statistical significance was set to α ≤ 0.05. Comparisons were made across all groups using two-way analysis of variance (ANOVA) with a *post-hoc* Tukey test with the following exceptions: Two-way ANOVA with repeated measures was used for comparisons of food intake over time, Student’s T-test (two-tailed) was used for comparisons in pSyn accumulation in the striatum between PFF injected PLX3397B and control rats, two-way ANOVA with *post-hoc* Tukey test comparisons in THir neurons in the SNpc were made within each brain hemisphere separately.

## Results

### Impact of microglial depletion during peak aggregation in the SNpc

#### Two months of Pexidartinib (PLX3397B) partially depletes microglia in both α-syn PFF and PBS injected rats

**Figure 2:**
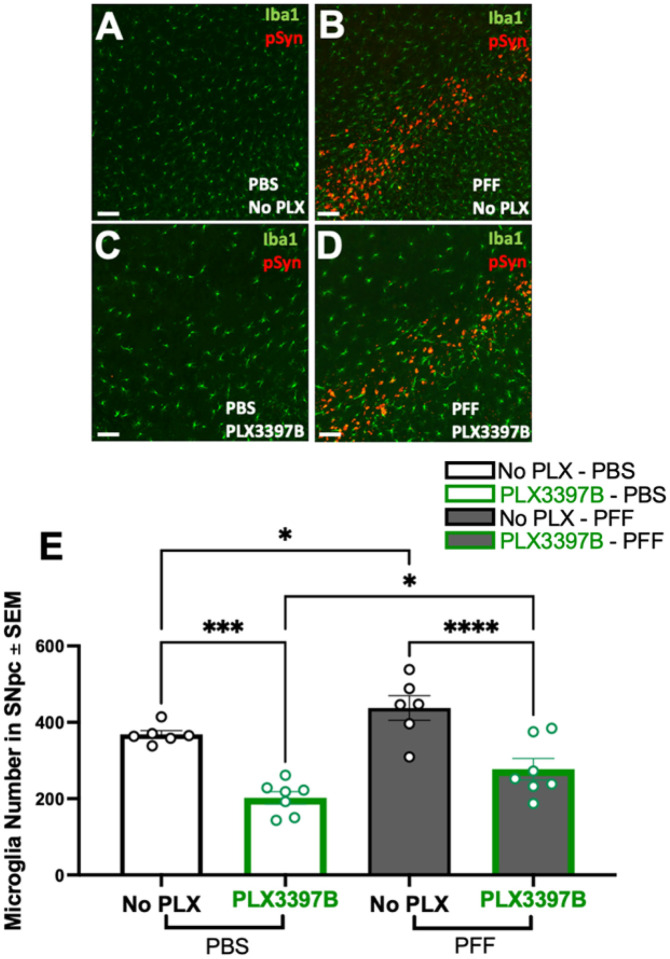
Inclusion-associated increases in microglia persist in the SNpc despite PLX3397B depletion of microglia. **A-D**: Ionized calcium binding adaptor molecule 1 (Iba1, green) and phosphorylated alpha synuclein at serine 129 (pSyn, red) immunofluorescence in the substantia nigra pars compacta (SNpc) two months post intrastriatal alpha-synuclein preformed fibril (α-syn PFF) or phosphate buffered saline (PBS) injection, with or without Pexidartinib (PLX3397B) treatment. **E**: Quantitation of Iba1 immunoreactive microglia in the SNpc in all treatment groups. PFF injected rats display significantly more microglia in the SNpc in both chow treatment groups. PLX3397B treatment resulted in significant microglial depletion in both PBS and PFF rats (p≤0.001). Black outline = no PLX3397B; green outline = PLX3397B; *p<0.04; ***p=0.0001; ****p<0.0001. Values represent the mean ± SEM. Scale bars in Panel A-D are 100μm.

PFF injected rats displayed substantial accumulation of pSyn within the SNpc ipsilateral to PFF injection as well as significantly more microglia compared to PBS rats, regardless of chow treatment (p<0.04, Figure 2A–E). Specifically, PFF injection was associated with ~19% and ~37% more microglia in the SNpc of control and PLX3397B chow rats, respectively. Treatment with Pexidartinib (PLX3397B; 600mg/kg) for 2 months led to a significant depletion of microglia within the SNpc in both PBS and α-syn PFF injected rats. PBS PLX3397B rats displayed 45% fewer microglia (p=0.001) and PFF PLX3397B rats displayed 36.6% fewer microglia (p<0.001) compared to the control fed rats in their respective surgical treatment groups (Figure 2E). These data suggest that inclusion-associated increases in microglia persist despite significant depletion of microglia due to 2 months of PLX3397B treatment.

#### Microglial depletion does not impact accumulation of pSyn aggregates in nigral neurons or early loss of TH phenotype

**Figure 3:**
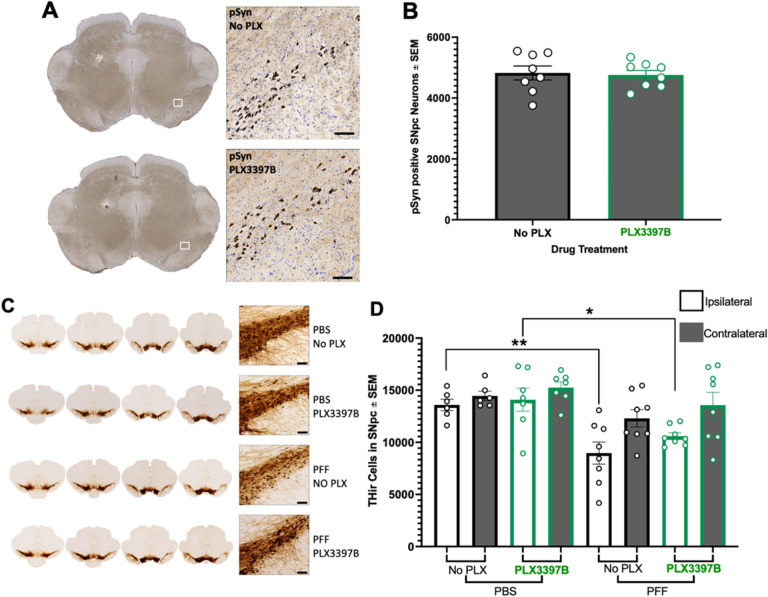
Microglial depletion does not impact pSyn aggregates or early loss of TH-immunoreactivity in the SNpc **A**: Phosphorylated alpha synuclein (pSyn) inclusions in the ipsilateral substantia nigra pars compacta (SNpc) two months post alpha synuclein preformed fibril (α-syn PFF) injection in both Pexidartinib (PLX3397B) and control fed rats. **B**: Quantification of pSynir neurons in the ipsilateral SNpc 2 months after α-syn PFF injection in control and PLX3397B rats. PLX3397B treatment had no impact on the number of pSynir neurons within the SNpc. **C**: Tyrosine hydroxylase immunoreactive (THir) neurons in the SNpc of PFF and control phosphate buffered saline (PBS) injected rats, with and without PLX3397B treatment. **D**: Quantification of THir neurons in the SNpc two months following injection. PFF injected rats possessed significantly fewer THir neurons in the ipsilateral SNpc as compared to the ipsilateral SNpc of PBS injected rats. No differences in THir neurons were observed due to PLX3397B treatment. Values represent the mean ± SEM. Black outline = no PLX3397B; green outline = PLX3397B; *p=0.0330; **p=0.0058. Scale bars in Panels A and C are 100 μm.

Intrastriatal injection of mouse α-syn PFFs results in peak pSyn accumulation in the ipsilateral SNpc at two months (17,21,22). In the present study we observed pSyn accumulation in the ipsilateral SNpc of PFF injected rats (Figure 2B, 2D, 3A) but not in PBS control rats (Figure 2A, C). PLX3397B treatment had no impact on the number of pSynir neurons within the SNpc of PFF rats (p>0.05, Figure 3B). PFF rats fed control chow possessed 4826 ± 229.3 pSyn containing neurons in the ipsilateral SNpc whereas PFF PLX3397B rats possessed 4760 ± 148.8.

We next examined whether PFF injection or PLX3397B treatment for two months impacted THir neurons in the SNpc. Utilizing identical PFF surgical parameters in rats we have previously observed ~0–25% loss of THir SNpc neurons at two months after PFF injection, however parallel neuronal counts revealed that this represents loss of TH phenotype in the absence of overt degeneration (17,38). In the present study, two months following PFF injection we observed a 24–33% reduction (p<0.04) in THir neurons in the ipsilateral SNpc as compared to the ipsilateral SNpc of PBS injected rats (Figure 3C, D) both with and without PLX3397B treatment. No differences in THir neurons were observed due to PLX3397B treatment (p>0.05). These results suggest that microglial depletion does not impact THir neurons in control rats, nor does it prevent the modest loss of TH phenotype associated with the aggregation phase of the PFF model.

#### Microglial depletion does not impact reactive microglia morphology or MHC-II expression associated with α-syn inclusions in the SNpc

**Figure 4:**
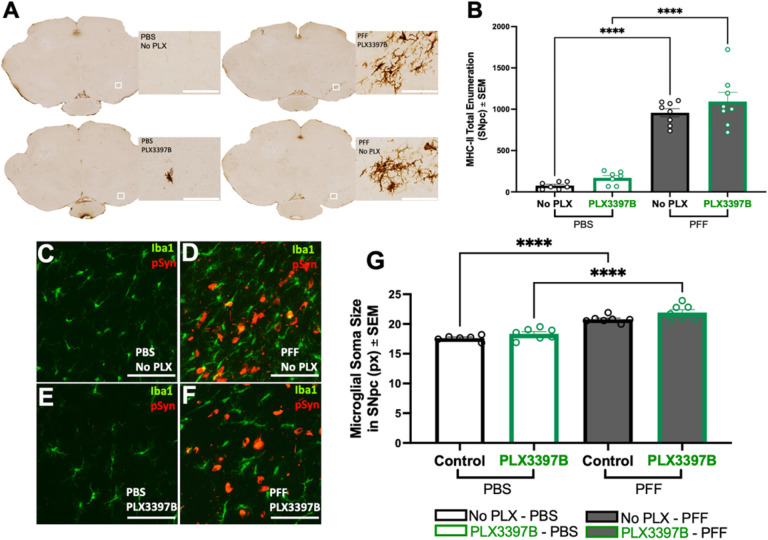
Localized inflammatory response to pSyn inclusions in the SNpc is preserved despite microglial depletion. **A**: Major histocompatibility complex II immunoreactive (MHC-IIir) cells in the ipsilateral SNpc of α-syn PFF or PBS injected rats with or without PLX3397B treatment. **B**: Quantification of MHC-IIir microglia in the ipsilateral SNpc demonstrates a significant increase in PFF compared to PBS rats at 2-months that is unaffected by PLX3397B treatment. **C-F**: Ionized calcium-binding adaptor molecule 1 (Iba1, green) and phosphorylated alpha-synuclein at serine 129 (pSyn, red) immunofluorescence in the ipsilateral substantia nigra pars compacta (SNpc) two months after intrastriatal alpha-synuclein preformed fibril (α-syn PFF) of phosphate buffered saline (PBS) injection, with or without Pexidartinib (PLX3397B). **G**:Quantification of Iba1 immunoreactivity (Iba1ir) microglia soma size demonstrates a significant increase following α-syn PFF injection as compared to PBS that is unaffected by PLX3397B treatment. Values represent the mean ± SEM. Black outline = no PLX3397B; green outline = PLX3397B; ****p<0.0001. scale bars in Panel A and C-F 100μm.

pSyn inclusions in the SNpc are associated with an increase in microglial soma size and a localized expression of MHC-II that correlates with α-syn inclusion load (21,38). In the present study, we observed numerous MHC-IIir microglia within the SNpc after intrastriatal α-syn PFF injection whereas very few MHC-IIir microglia were observed in PBS control rats (Figure 4A). Significantly more MHC-IIir microglia were observed in both PFF control and PFF PLX3397B SNpc compared to PBS injected rats (p<0.0001, Figure 4B). No significant differences were observed in the number of MHC-II-ir microglia due to PLX3397B treatment (p>0.05, Figure 4B). Rats with nigral pSyn inclusions exhibited significantly larger microglial soma size in the ipsilateral SNpc compared microglia in the ipsilateral SNpc of PBS control rats, regardless of PLX3397B treatment (p<0.0001, Figure 4C–G). In general, Iba-1 immunoreactive microglia were 15–20% larger in the SNpc of PFF injected rats. No significant differences in microglial soma size were observed within PBS or PFF treatment groups due to PLX3397B (p>0.05). Collectively, these results suggest that despite significant depletion of microglia, the localized inflammatory response to pSyn inclusions in the SNpc is preserved.

### Impact of microglial depletion during nigrostriatal degeneration phase

#### Six months of Pexidartinib (PLX3397B) partially depletes microglia in both α-syn PFF and PBS injected rats

**Figure 5:**
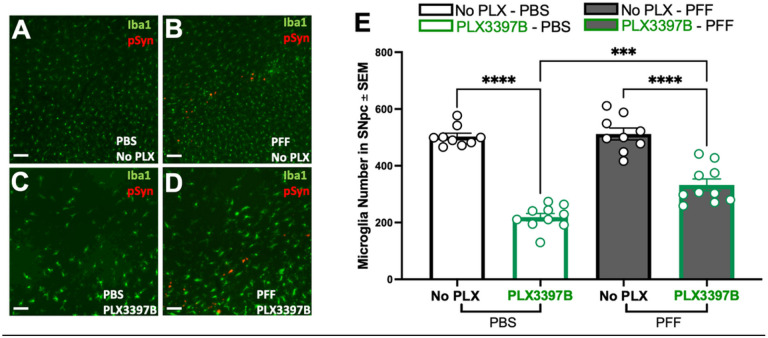
PLX3397B is less effective in microglial depletion during nigrostriatal degeneration. **A-D**: Ionized calcium-binding adaptor molecule 1 (Iba1, green) and phosphorylated alpha-synuclein (pSyn, red) immunofluorescence in the substantia nigra pars compacta (SNpc) six months following intrastriatal alpha-synuclein preformed fibril (α-syn PFF) or phosphate buffered saline (PBS injection), with or without PLX3397B. Modest accumulation of pSyn immunoreactive neurons in the ipsilateral SNpc is evident following α-syn PFF injection. **E**. Quantitation of Iba1 immunoreactive microglia in the SNpc in all treatment groups. Six months of PLX3397B treatment resulted in significant microglial depletion in both PBS and PFF rats. PFF PLX3397B rats display significantly more microglia compared to PBS PLX3397B rats. Values represent the mean ± SEM. No PLX3397B = black outline, PLX3397B = green outline. ****p<0.0001 ***p=0.0001. Scale bars in Panels A-D are 100μm.

PFF injected rats displayed modest accumulation of pSyn within the SNpc ipsilateral to PFF injection, however microglia number was not increased due to PFF injection (p>0.05, Figure 5A–E). Similar to the effect of 2 months of PLX3397B treatment, 6 months of PLX3397B led to a significant depletion of Iba-1 immunoreactive microglia in both PBS and α-syn PFF injected rats (Figure 5E, p<0.001). PBS PLX3397B rats displayed 56% fewer microglia and PFF PLX3397B rats displayed 36% fewer microglia compared to control fed rats in their respective surgical treatment groups. Further, PLX3397B PFF rats possessed significantly more microglia than PLX3397B PBS rats (51% increase, p=0.001). Our results confirm successful depletion of microglia using PLX3397B over the 6-month interval and suggest that during PFF-induced nigrostriatal degeneration PLX3397B is less effective in microglial depletion.

#### Microglial depletion does not impact pSyn inclusion triggered degeneration of nigral dopamine neurons

**Figure 6:**
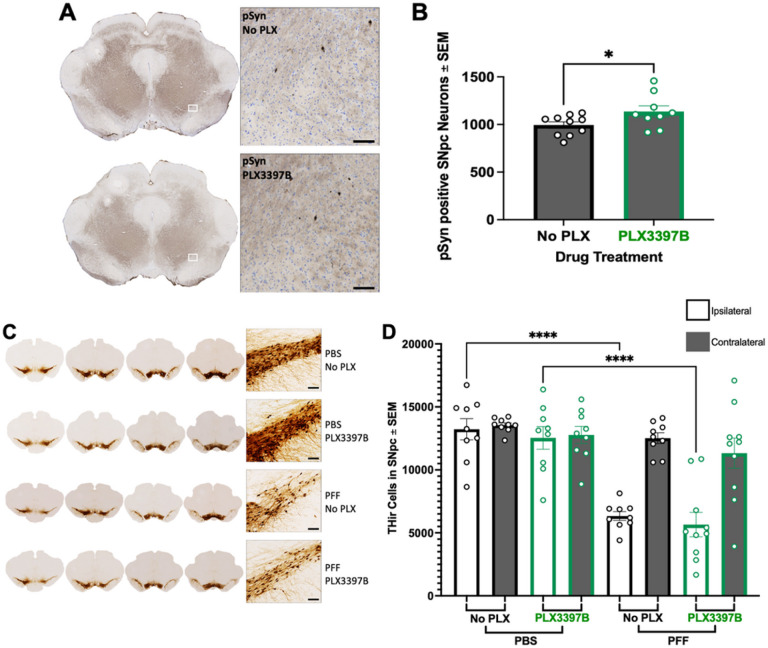
Microglial depletion does not impact degeneration of nigrostriatal dopamine neurons following α-synuclein preformed fibril injection. **A**: Phosphorylated α-syn (pSyn) inclusions in the ipsilateral substantia nigra pars compacta (SNpc) six months post alpha-synuclein preformed fibril (α-syn PFF) injection in both PLX3397B and control fed rats. **B**. Quantification of pSyn containing neurons in the ipsilateral SNpc in rats 6 months after α-syn PFF injection. Significantly fewer pSyn SNpc neurons are observed in PFF rats fed PLX3397B. **C**: Tyrosine hydroxylase immunoreactive (THir) neurons in the SNpc of PFF and control phosphate buffered saline (PBS) injected rats, with and without 6 months of PLX3397B treatment. **D**. Quantification of THir neurons in the SNpc 6 months following surgery. PFF injection resulted in significant loss of THir SNpc neurons in both PLX3379B and control fed rats. Values represent the mean ± SEM.****p<0.0001 *p<0.05. PLX3397B = green outline, no PLX3397B = black outline. Scale bars in Panels A and C are 100μm.

Our previous work has demonstrated that few pSyn inclusions remain in the SNpc 6 months following PFF injection due to the loss of the SNpc neurons that were initially seeded (17,21). In general, the number of pSyn immunoreactive SNpc neurons observed at 6 months represents 10–20% what is observed during the peak 2-month aggregation phase (17). In the present study we similarly observed an approximate 80% reduction in pSyn immunoreactive neurons in the SNpc at 6 months when compared to 2 months post α-syn PFF injection (p<0.0001). A modest yet significant increase in pSynir SNpc neurons was observed in PFF PLX3397B rats compared to PFF rats fed control chow (p=0.0470; Figure 6A,B). We also evaluated the impact of PLX3397B on pSyn accumulation in the striatum, a structure in which pSyn accumulation is abundant at the 6-month time point (17). No significant differences were observed in pSyn accumulation in the striatum of PFF PLX3397B rats compared to PFF control chow rats (p>0.05, Supplemental Figure 3). These data suggest that 6 months of PLX3397 treatment results in little to no impact on pSyn accumulation following PFF injection.

Previous rat PFF model studies using identical surgical parameters reveal significant loss of ipsilateral SNpc THir neurons 5–6 months post intrastriatal α-syn PFF injection that parallels frank neuronal loss (17). In the present study, 6 months following surgery, we observed a 52–55% reduction in THir neurons in the ipsilateral SNpc of PFF rats as compared to the ipsilateral hemisphere of PBS injected rats (p<0.0001), both with and without PLX3397B treatment (p<0.0001, Figure 6C,D). Specifically, the ipsilateral SNpc of PFF rats fed control chow possessed 6333 ± 349.5 THir neurons whereas the ipsilateral SNpc of PBS rats fed control chow possessed 13221 ± 838.1 THir neurons. The ipsilateral SNpc of PFF PLX3397B rats possessed 5658 ± 967.2 THir neurons compared to 12536 ± 896.8 THir neurons in the ipsilateral SNpc of PBS PLX3397B rats. No significant differences were observed in ipsilateral SNpc THir neurons in PFF rats due to PLX3397B (p>0.05). These results suggest that microglial depletion does not impact the loss of THir SNpc neurons during the degeneration phase of the PFF model.

#### Long term microglial depletion results in increased microglia soma size and emergence of MHC-II expression in areas outside the SNpc

**Figure 7:**
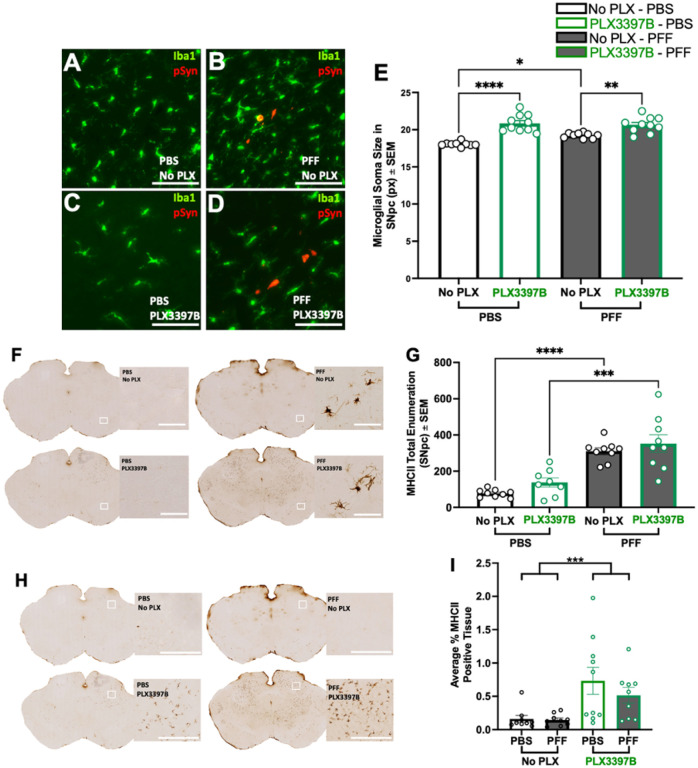
Long-term microglial depletion increases microglial soma size and extranigral major histocompatibility complex II expression. **A-D**: Ionized calcium-binding adaptor molecule 1 (Iba1, green) and phosphorylated alpha-synuclein (pSyn, red) immunofluorescence in the ipsilateral substantia nigra pars compacta (SNpc) six months following intrastriatal alpha-synuclein preformed fibril (α-syn PFF) or phosphate buffered saline (PBS) injection, with or without PLX3397B. Modest accumulation of pSyn immunoreactive neurons in the ipsilateral SNpc is evident following α-syn PFF injection. **E**. Quantification of microglial soma size demonstrates a significant increase associated with PLX3397B. In rats not fed PLX3397B, but not in PLX3397 rats, α-syn PFF injection is associated with increased microglial soma size. **F**. Major histocompatibility complex II immunoreactive (MHC-Iiir) microglia in the ipsilateral SNpc of PFF and PBS injected rats, with and without six months of PLX3397B treatment. **G**. Quantification of MHC-IIir microglia in the SNpc demonstrates a significant degeneration-associated increase as compared to PBS injected rats at 6-months that is not affected by PLX3397B treatment. **H**. MHC-II expression outside of the SNpc in both α-syn PFF and PBS rats after six months PLX3397B treatment. **I**. Quantification of MHC-II-ir expression in the midbrain parenchyma revealed a significant increase associated with long term PLX3397CB treatment. Values represent the mean ± SEM. Black outline = no PLX3397B, green outline = PLX3397B. ****p<0.0001 ***p=0.001, ** p<0.01, *p<0.05. Scale bars in Panels A-D and F are 100μm. scale bars in Panel H are 500 μm.

Analysis of the microglial soma size at six months revealed that PFF injected rats possessed significantly larger microglia in the SNpc compared to PBS control rats regardless of PLX3397B treatment (p=0.0194, Figure 7A–E). Further, six months of microglial depletion led to a significant increase in microglial soma size in both PBS and PFF injected animals (p<0.001). We next analyzed the number of MHC-IIir microglia in the SNpc ipsilateral to injection. MHC-IIir microglia peak in abundance in the SNpc 2 months after intrastriatal PFF injection, in immediate proximity to pSyn inclusions (21). Although the number of MHC-IIir microglia decrease in abundance over time, MHC-IIir microglia remain elevated compared to controls during the degenerative phase at 6 months (21). In alignment with these earlier observations, in the present experiment we observed a significant decrease in the number of MHC-IIir microglia in the SNpc of PFF injected rats at six months compared to two months (p<0.0001), representing a reduction of approximately 70%. Despite the reduced population of MHC-IIir microglia, we observed a significant increase in MHC-IIir microglia in PFF rats compared to controls, in both PLX3397B treated (p=0.0001) and untreated (p<0.0001) groups (Figure 7F, G). Specifically, PFF control chow rats possessed 302% more MHC-IIir microglia than PBS control chow rats, whereas PFF PLX3397B rats possessed 214% more MHC-IIir microglia than PBS PLX3397B rats. Within surgical treatment groups, no significant differences were observed in the number of MHC-IIir microglia in the SNpc due to PLX3397B treatment (p>0.05, Figure 7G). Further, in rats that received PLX3397B chow (both PFF and PBS) we also noticed MHC-II expression in the mesencephalon outside the nigral region (Figure 7H). Quantification of MHC-II expression in the extranigral mesencephalon revealed a significant increase associated with long term PLX3397CB treatment (p=0.0006; Figure 7I). Collectively, these results suggest that despite significant microglial depletion, the localized inflammatory response to nigral degeneration normally observed following PFF injection is preserved. Further, long term microglial depletion may produce an enhanced proinflammatory phenotype in remaining microglia.

## Discussion

Imaging and histological studies provide support for the presence of ongoing neuroinflammatory processes in PD (12,15,16,39–41). The ability to attenuate inflammatory processes through microglial depletion has yielded mixed results in both AD (Tau; (30)) and PD (MPTP;(42)) animal models. In some studies, microglial depletion has led to the exacerbation of neurodegeneration (43–45) whereas in others neuroprotection is observed (42,46). Previous studies using CSF1R inhibitors in mice employed dosing strategies that resulted in near complete microglial depletion (~90%) (27,46,47). However, microglia play many roles in maintaining healthy homeostasis in the brain (3,4,48)and thus complete microglia depletion may not be a safe therapeutic strategy. Therefore, in the present study we employed a PLX3397B dosing strategy that elicited partial (~40%) microglial depletion in the SNpc. Our results demonstrate that partial microglial depletion does not prevent α-syn aggregation in the SNpc or the striatum, attenuate the inflammatory response to aggregation or degeneration, or prevent nigral degeneration following intrastriatal PFF injection.

Our previous studies have revealed that microglia react to the aggregation and degeneration phases of the rat α-syn PFF model in a consistent, measurable manner (21,22,38). During the peak aggregation phase in the SNpc at 2 months, microglia increase in number, soma size and MHC-II expression. The MHC-II response of microglia to intraneuronal pSyn aggregates is heterogeneous, limited to a subpopulation of microglia within the immediate vicinity of the SNpc inclusions. The heterogeneity of inflammatory responses within individual microglia (49) and between different brain regions (4) has been well-documented. The number of MHC-IIir microglia positively correlates to the number of pSyn immunoreactive SNpc neurons and is markedly decreased during the nigral degeneration phase (21). In the present study, microglial depletion with PLX3397B attenuated the increase in microglia associated with aggregation and degeneration in the SNpc, as would be expected, but the soma size of the remaining microglia increased in rats that received PLX3397B for 6 months. Further, the MHC-IIir microglial subpopulation was not impacted by microglial depletion. We had initially hypothesized that with ~40% depletion of microglia we would observê40% reduction in MHC-IIir microglia with PLX3397B. The maintenance of the pSyn inclusion associated MHC-IIir microglia subpopulation, despite significant microglial depletion, suggests that the remaining microglia maintain the capacity to mount a similar proinflammatory response. This finding is not unique to this study, as other microglia depletion studies have shown similar maintenance or an increase in inflammatory responses when general microglial populations are depleted (27,30,50) along with increases in adaptive immune cells within the brain (51). Our findings point to a need to understand the full phenotype of the inclusion associated MHC-IIir microglial subpopulation that is maintained despite significant general microglial depletion.

The approach of microglia repopulation as a therapeutic strategy in order to “reset” microglia has been recently proposed with the goal of exchanging dysfunctional with functional microglia. However, the results from repopulation studies vary (28,47) and suggest that repopulation comes from the remaining microglia. Our data suggests a microglia repopulation strategy would not be beneficial, and that the inflammatory response to pSyn inclusions and nigrostriatal degeneration would be maintained.

Our study is unique in that the microglial depletion was sustained for a period of 6 months, whereas most previous microglial depletion studies use much shorter depletion intervals (7–28 days (28,29,43,47,50). We observed evidence of a more pronounced inflammatory state in our 6-month microglial depletion study compared to our 2-month microglial depletion study. Specifically, after 6 months of microglial depletion, microglia soma size was increased, even within control PBS injected rats. Further, after 6 months of microglial depletion we observed MHC-IIir cells in multiple brain regions, and also in control rats. Normally, except for border associated macrophages (52,53), MHC-II immunoreactive cells are not often observed in uninjured brain regions in control rats. The increased MHC-II expression we observe with long term microglial depletion may be attributable to microglia or to infiltrating monocytes, border associated macrophages or perivascular macrophages (24,54–56). Future investigation is required to ascertain the identity of the cells that respond to microglial depletion with upregulated MHC-II expression.

## Conclusions

Inflammatory microglia may contribute to PD progression and microglial based inflammation has been under investigation in order to identify therapeutic targets. One limitation of the present study is that the response of other cell types (peripheral macrophages, astrocytes, adaptive immune cells) to microglial depletion was not examined. Previous studies have indicated that near complete microglial depletion can impact astrocytes and adaptive immune cells (30). Another limitation of the present study is that the magnitude of microglial depletion was not that which has been previously achieved in mouse studies (~90%) (27,46,47). It is possible that near complete levels of microglial depletion may have yielded different outcomes. Despite these limitations, the present study suggests that partial microglial depletion may not be an effective, disease-modifying approach for PD and may instead induce a heightened proinflammatory state in remaining microglia.

## Data Availability

The datasets supporting the conclusions of this article are available from the corresponding author upon reasonable request.

## Abbreviations

α -syn: Alpha-synuclein
AAALAC: Association for Assessment and Accreditation of Laboratory Animal Care
ANOVA: Analysis of variance
DA: Dopamine
DAPI: 4’,6-Diamidino-2-Phenylindole, Dihydrochloride
CSF: Cerebral Spinal fluid
CSF1R: Colony stimulating factor 1 receptor
HLA-DR: Human leukocyte antigen-antigen D related
IACUC: Institutional Animal Care and Use Committee
Iba1: Ionized calcium binding adaptor 1
IF: Immunofluorescence
IHC: Immunohistochemistry
ir: Immunoreactive
MHC-II: Major histocompatibility complex II
mPFFs: Mouse preformed fibrils
NGS: Normal goat serum
PB: Phosphate Buffer
PBS: Phosphate buffered saline
PD: Parkinson’s disease
PFA: Paraformaldehyde
PFFs: Preformed fibrils
PLX3397B: Pexidartinib 3397B
pSyn: Phosphorylated alpha-synuclein at SER 129
SN: Substantia nigra
SNpc: Substantia nigra pars compacta
STR: Striatum
TBS: Tris buffered saline
TH: Tyrosine hydroxylase
Tx-100: Triton X-100
